# Smart and Age-Friendly Cities in Romania: An Overview of Public Policy and Practice

**DOI:** 10.3390/ijerph17145202

**Published:** 2020-07-18

**Authors:** Loredana Ivan, Dorin Beu, Joost van Hoof

**Affiliations:** 1 Department of Communication, National University of Political Studies and Public Administration, Bulevardul Expoziţiei 30A, 012102 Bucharest, Romania; loredana.ivan@comunicare.ro; 2 Faculty of Building Services Engineering, Technical University of Cluj Napoca, Strada Memorandumului 28, 400114 Cluj-Napoca, Romania; dorin.beu@insta.utcluj.ro; 3 Faculty of Social Work and Education, The Hague University of Applied Sciences, Johanna Westerdijkplein 75, 2521 EN Den Haag, The Netherlands; 4 Institute of Spatial Management, Faculty of Environmental Engineering and Geodesy, Wrocław University of Environmental and Life Sciences, ul. Grunwaldzka 55, 50-357 Wrocław, Poland

**Keywords:** age-friendly cities, smart cities initiatives, smart city, Romania, public policies on smart cities, evaluating smart cities initiatives, older people

## Abstract

The role of smart cities in order to improve older people’s quality of life, sustainability and opportunities, accessibility, mobility, and connectivity is increasing and acknowledged in public policy and private sector strategies in countries all over the world. Smart cities are one of the technological-driven initiatives that may help create an age-friendly city. Few research studies have analysed emerging countries in terms of their national strategies on smart or age-friendly cities. In this study, Romania which is predicted to become one of the most ageing countries in the European Union is used as a case study. Through document analysis, current initiatives at the local, regional, and national level addressing the issue of smart and age-friendly cities in Romania are investigated. In addition, a case study is presented to indicate possible ways of the smart cities initiatives to target and involve older adults. The role of different stakeholders is analysed in terms of whether initiatives are fragmentary or sustainable over time, and the importance of some key factors, such as private–public partnerships and transnational bodies. The results are discussed revealing the particularities of the smart cities initiatives in Romania in the time frame 2012–2020, which to date, have limited connection to the age-friendly cities agenda. Based on the findings, a set of recommendations are formulated to move the agenda forward.

## 1. Introduction

All over the world, societies are ageing and older people become increasingly visible in daily living. According to the Organisation for Economic Cooperation and Development (OECD) [1], the population share of those adults aged 65 years and over is expected to rise to 25.1% in 2050 across its member states. A similar pattern can be observed for Romania, a European Union member state which also has an intention to become an OECD member state and whose membership request is under consideration by the OECD Council. Cities in particular have large numbers of older inhabitants; over 40% of all older people live in urban areas. The relationships between ageing populations and urban change, in conjunction with the need to develop supportive urban communities, are a cause of issues and concerns for public policies and practise [2].

The understanding of these relationships led to the emergence of so-called age-friendly cities [3,4,5,6]. There are eight domains of an age-friendly city, namely Social participation; Communication and information; Civic participation and employment; Housing; Transportation; Community support and health services; Outdoor spaces and buildings; Respect and social inclusion. The WHO proposed that policies, services, and structures in an age-friendly city, which are related to the physical and social environment, are designed to support and enable older people to “age actively”. In other words, to live in security, enjoy good health, and continue to participate fully in society [3,6,7,8]. 

Within the framework of age-friendly cities and communities, technology does not play an explicit role [9], but it needs to be noted that in the daily living of older people, technology is all around and cannot be ignored as a major source of support for activities, stimulation of social participation and the provision of care [10,11,12,13]. Given the numerous technologies available at present and used by citizens, it is important to explore how such technologies can be used and deployed in the home and across different communities in order to benefit the citizens in the respective age-friendly communities including those communities that are not categorised as age-friendly by the WHO [6,9,10,14]. The new smart age-friendly ecosystem framework proposed by Marston and van Hoof [9] considers the rapid pace in which technology develops but also to ensure all citizens in society are represented, and intersects with the current trends in the domain of smart cities.

Albino et al. [15] attempted to clarify the meaning of smart cities through a literature review, and found that it first emerged in the 1990s. They concluded that it is a multifaceted phenomenon, which includes, amongst others, qualities of people and communities as well as ICTs. There seems to be a lack of universality, both in its definition and in measures of performance [15,16]. Some of the definitions include references to healthcare, for instance, that a smart city is prepared to provide conditions for a healthy and happy community under the challenging conditions that global, environmental, economic, and social trends may bring. Or, the use of smart computing technologies to make the critical infrastructure components and services of a city, including healthcare, more intelligent, interconnected, and efficient [15]. In this paper, a smart city is an urban area that utilises and deploys various electronic Internet of Things devices and sensors [12], which have the ability to collect data and utilise the data in an attempt to provide efficient and smarter resources to residents and communities. A wide array of data can be collected from public and private sources, which can be processed for the better good of society [17]. In relation to advancing age-friendly interventions, the WHO touches upon the collaboration with transnational (city) networks, such as smart cities networks, for which addressing ageing will help advance their strategic priorities [9]. Podgórniak-Krzykacz et al. [18] also called for smart cities to seek to ensure meeting the needs of older citizens and promoting solutions tailored to their computer literacy, digital skills, and perception capabilities. A similar trend was also seen by Woolrych et al. [19] in relation to emerging technologies for the support of health and independence of senior citizens. In their study, many senior citizens were willing to use a wide range of technologies in the context of age-friendly smart cities. In a study on age-friendly city development in South Australia, Zaman and Thornton [20] identified a large set of priority indicators. One of the topics raised is the provision of training for older people on the latest technologies. Additionally, Gudowsky et al. [21] and Righi et al. [22] stated that smart cities need to adapt to ageing societies and that great hopes are projected on technology to support solutions for urban ageing. 

**Figure 1:**
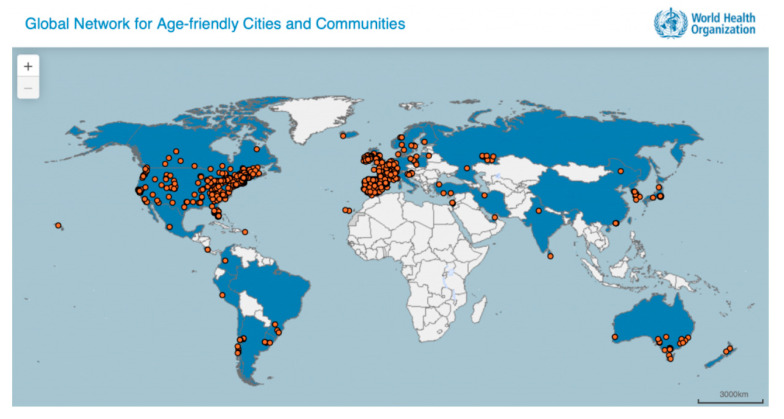
No city in Romania has yet joined the Global Network for Age-friendly Cities and Communities (status quo July 2020) [23].

A growing number of cities and communities worldwide are striving to better meet the needs of their older residents. The WHO Global Network for Age-friendly Cities and Communities was established to foster the exchange of experience and mutual learning between cities and communities worldwide (Figure 1). Cities and communities in the network are of different sizes and are located in different parts of the world. Their efforts to become more age-friendly take place within very diverse cultural and socioeconomic contexts. What all members of the Network do have in common is the desire and commitment to promoting healthy and active ageing and a good quality of life for their older residents. Romania does not have any cities or communities that are members of the WHO Global Network for Age-friendly Cities and Communities. At the same time, there are numerous smart city initiatives in the country, which may or may not intersect with the age-friendly cities movement and its strategic agenda.

The current study investigates smart city initiatives at the local, regional, and national level in Romania aiming to answer the following research questions:

Research Question 1: *What are the main characteristics of smart city initiatives in Romania during the past eight years*? The time period 2012–2020 is used as a time frame after exploring the moment such initiatives started to develop to a large scale, in several Development Regions around the country. The goal is to investigate whether the initiatives were mainly national or local, private or public, coherent or sporadic, short-term-oriented or long-term-oriented; what was the role of the industry and of the private operators, the type of projects (strategic, policy-oriented, concrete actions, etc.), and also what was the role of the transnational organisations in financing and promoting such initiatives?

Research Question 2: *To what extent do the smart cities initiatives in Romania, for the past eight years, have an age-friendly component*? This question investigates the extent to which the large-scale smart city projects developed in Romania had frail and vulnerable groups of people as an explicit objective, for instance, in terms of access to urban facilities and urban life in general. The main focus is on the older population and whether such initiatives took the older population into consideration in developing smart cities initiatives.

The paper starts with background information on the country of Romania and key cities. This is followed by an overview of the methodology and the results of the social document analysis on existing smart city initiatives and practices from Romania. Subsequently, the results are discussed revealing the particularities of the smart cities initiatives in Romania, in the time frame 2012–2020. This is followed by a conclusion and recommendations section for future steps that Romania could take to move the agenda forward, and how to connect the smart cities’ and age-friendly cities’ agendas for the future development of urban centres in Romania.

## 2. Outline of Romania

**Figure 2:**
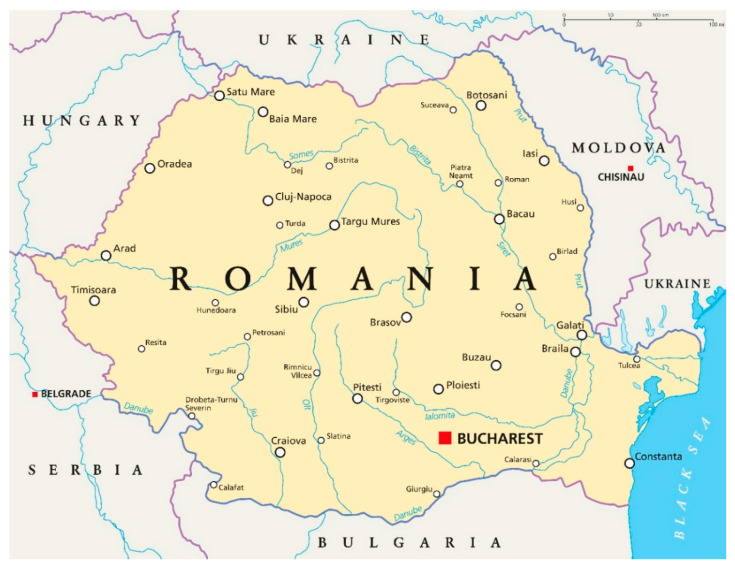
Map of Romania and its main population centres. Source: https://www.shutterstock.com/nl/image-vector/romania-political-map-capital-bucharest-national-321930074.

**Figure 3:**
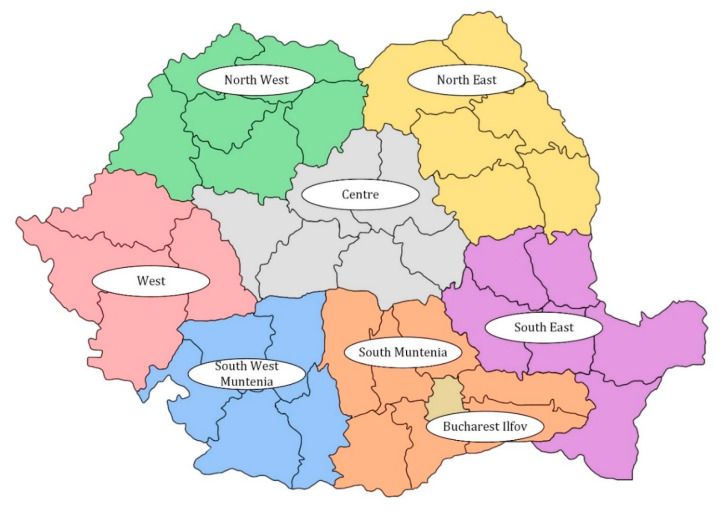
Map of Romania and its Development Regions. Taken and adapted from source: https://en.wikipedia.org/wiki/Development_regions_of_Romania#/media/File:Regiuni_de_dezvoltare.svg.

The Republic of Romania is divided into three major regions: Walachia (Southern part, with Bucharest the capital of Romania as the main city), Transylvania (Western part, with Cluj-Napoca and Brașov as main cities) and Moldavia (Eastern part—with Iași as the main city), and as well as some other minor regions (Figure 2). The Carpathian mountain range is the main natural barrier dividing these major regions. The territorial and administrative organisation of Romania is based on Law 2/1968, which is still valid today. There are seven so-called Development Regions (North-West, West, Centre, North-East, South-East, South-West, and South) plus the Bucharest and Ilfov Region (Figure 3). Romania’s demographics are split between Romanian nationals (22,204,507 in 2019) and actual residents (19,414,458 in 2019) due to the emigration of the population to other European Union member states (Table 1). The last census was held in 2011 when 21.4 million people were expected to live in the country. The final outcome of the census was a population of 20.1 million people, as many citizens are working abroad without a work permit, or by returning every three or six months from working abroad. Officially, between 1990 and 2018, 577,555 persons have emigrated permanently, but the actual number of people working abroad remains unclear. Some journalists mention a potential decline of the Romanian population by as much as 30% in 2050 based on international reports [24]. 

According to official figures from the National Statistics Institute [26], where data is available for the years 1992 to 2019, there is a decline of around 4% of the population (Table 2). The percentage of the urban population has risen from 53.9 to 56.4%. When it comes to the ageing of the population, the percentage of older people has risen from 10.9% in 1992 to 16.5% in 2019 (for people over 85 years from 0.6 to 1.8%, Table 3 and Table 4). This increase is mainly due to urbanisation, and the availability of better healthcare services. 

Take, for example, the city of Cluj-Napoca. In this city, there is a slight increase of the population with around 4%, but a major increase of the older population from 8.65% in 1992 to 17.14% in 2019 (Table 4). Like many Romanian cities, a large number of people moved from the rural area to towns during communist times, when there was a high demand for a labour force in the industry. This also explains the higher percentage of the older people in the countryside. Since 2000 a lot of new buildings have been developed in the area around Cluj-Napoca. Floreşti, a village in the suburban area of Cluj-Napoca, has seen its population increase from 5574 inhabitants in 1992 to 39,652 in 2019, which is more than seven times as much. 

According to the OECD [25], the life expectancy of Romanians has increased from 71.2 in 2000 to 75.3 years in 2017, with a high gender gap of 7.4 years (71.7 years for men and 79.1 for women). The most important cause of death remains ischaemic heart disease (three times higher than in the European Union as a whole) and stroke. The proportion of people aged 65 years and over reported to be in good health is 23% (compared with a European Union average of 41.4%). A total of 46% of the population aged 65 years and over have one or more chronic diseases, and 31% of this population reports some limitations in their activities of daily living.

In 2017, Romania spent €1029 per capita for health and most of this amount was spent on hospital care (42%) and pharmaceuticals and medical devices (27%). There is a shortage of physicians and nurses in Romania, which is mainly due to the large-scale emigration of skilled workers to countries that offer higher salaries. There are also additional challenges concerning the availability of general practitioners (GPs) in Romania: 328 communities (villages) have no GP available. A total of 559,611 persons, or 2.52% of Romanians, have no GP in the village where they live. In a total of 1414 cities and villages, there is a shortage of 2187 GPs. Only 271 cities have sufficient or even excess GP capacity, such as Bucharest and other university cities [27].

In each of the Romanian cities, there is a Department of Social Assistance, which includes departments like child and family protection. This department also takes care of the provision of assistance for older people. The service was initiated after 1990, mainly for solving the problem of abandoned children in Romania. It is currently in a slow transition towards providing services for an ageing population. The Department offers services such as daycare and activities in activity centres for older people, but currently, no projects are running to support the use of technology by older people.

### Smart and Sustainable Housing

According to the Buildings Performance Institute Europe (BPIE) [16], 47.5% of the dwellings are in the rural area (95% unfamiliar houses) and in the urban area, 72% of the dwellings are in large blocks of flats, with an average of 40 apartments per block. Additionally, according to BPIE [28], the average surface of a dwelling is 55 m^2^, with a majority of them built between 1960 and 1990. As the comfort standard of homes built during this period was low, there is a clear need for investment to update the quality of living and the quality of the home itself. According to Eurostat [29], the housing market in Romania is largely in private hands. A total of 96.8% of people live in owner-occupied dwellings, of which a mere 47% are overcrowded. This limits the use of European, national or local funding schemes for the improvement of homes. In Romania, the use of European funds is limited to improvements of the outer shells of buildings, for instance, adding or improving thermal insulation, and installing new insulating windows. National programmes can be used to install photovoltaic panels. At present, there are no (structural) subsidies for upgrading existing homes with smart home technologies. When it comes to new residential developments, there is a local sustainable certification system called Green Homes, developed by Romanian Green Buildings Council—RoGBC [30], which encourages sustainable and smart housing. According to RoGBC, a total of 10,905 new apartments in 44 projects are Green Homes certified [30]. The domains of this certification that can be classified as age-friendly are as follows:

- Entrances (installation of ramps, lighting, and automated doors for wheelchair access);
- Lighting (better lighting for safer homes, avoidance of glare, dimming options and night orientation lighting from the bedroom to toilet, motion sensors in circulation areas);
- Circulation space (possibility for turning a wheelchair in all spaces of the home);
- Accommodation of mobility devices (wheelchairs);
- Toilet and bathroom walls (firm fixing and grab bars/rails);
- Location of control services (height band from 450 mm to 1200 mm above the floor level);
- Flooring materials in common spaces (soft and resilient materials to avoid falls and gentler underfoot);
- Installation of handrails (both sides of a wall at high and low levels);
- Way-finding in common areas (use of different colours and textures and Braille signage);
- Installation of an intercom which is easy-to-use with visual intercom for people with a hearing impairment.

All the features mentioned above are part of a set of hardware solutions for an age-friendly home, which can be supplemented by smart home solutions. At present, there is no national programme intended for the construction of smart homes and there are no regulations for the design of age-friendly homes. 

## 3. Methodology

**Figure 4:**
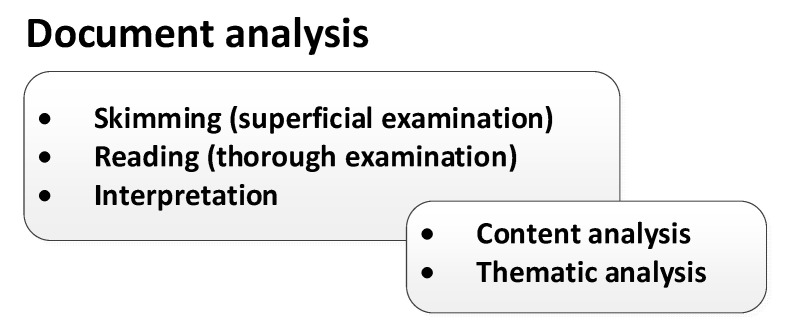
The process of document analysis [31].

Document analysis [31], which is based on keyword clustering, was used as a method to evaluate the current state-of-the-art of smart city programmes and action plans in Romania (Figure 4). In addition, it was also investigated whether the smart city strategies and solutions were age-inclusive or had an age-friendly component. By clustering, documents based on the criteria of keyword similarity were grouped. Two keywords were used to cluster the documents: “smart city” and “age-friendly city” (in Romanian smart-city and age-friendly are neologisms, used as such in the current language). The frequencies of these two keywords were studied in the documents and potentially for the co-occurrence of the two terms.

### 3.1. Corpus of Analysis

A variety of policy papers, official documents, and websites presenting different initiatives both from the public institutions and from the industry, pieces of legislation and presentations in local conferences on the topic of smart city, were included. Programmatic documents and concrete projects were selected, which contained a set of measures impacting the community at the local, regional or national levels, affecting the lives of a sufficient number of people, and containing more than one single output, with a duration of at least one year.

In total, 53 documents were selected after searching on the main news portal in Romania [32], using the keywords: “smart city” and “age friendly city” and on the dedicated websites of the public institutions and professional organisations. These websites included those of The Romanian Association for Smart City and Mobility (RSCMA); The Ministry of Energy, The Ministry of Waters and Forests; The Chamber of Commerce and Industry of Romania; The Ministry of Regional Development and Public Administration of Romania; The Ministry of Telecommunication; and The Ministry of Health. Documents were selected for the time frame of 2012–2020, based on the fact that the year 2012 marks the year in which the first programmes labelled as “smart city” emerged in Romania. Once we identified the main cities in which such programmes were deployed (namely, Alba Iulia, Bucharest, Brașov, Baia Mare, Cluj-Napoca, Craiova, Iași, Suceava, Vaslui, Oradea, and Timișoara), the respective websites of these municipalities were also searched, as the particular municipality or local administration had a role in all local or regional initiatives that were encountered in this search. 

### 3.2. Coding Procedure

In the first phase, initiatives belonging to the same programme and having the same stakeholders were grouped separately. In such cases, the main objective of the programme and the key action plan were used for clustering the documents. Programmatic documents were grouped as well (as, for example, national strategies), separately from the concrete projects containing a set of measures impacting the community at the local, regional, or national level. 

In the final stage, documents were selected taking into account the following criteria: whether they affect the lives of a sufficient number of people, and contain more than one single output, with a duration of at least one year. In total, there were 30 initiatives, which were coded in line with the research questions. An axial coding system was used to code the documents on the following categories: (2) the organisation which initiated the programme and the key stakeholders (1) the type of action (local, regional, national); (3) the role of private/public sector; (4) type of document (programmatic documents, strategies, concrete actions, publicity, training, or other types of initiative); (5) the main objectives; (6) the age-friendly component—implicit or explicit; (7) the role of the industry (for example, in suggesting solutions/realising the actions); (8) the role of a transnational bodies/organisations—for example, in advising, promoting, financing, launching a particular initiative. The year (of the time frame) of the initiatives included in the analysis were recorded. 

## 4. Results

This section presents the main characteristics of smart city initiatives in Romania in 2012–2020. The results from the coding process are presented in detail in Appendix A. The following subsections present the findings from this table in extenso. These subsections deal with (1) The regional disparities, and lock on inter-regional cooperation; (2) The governance; (3) The type of project, and the main objectives, (4) The role of transnational organisations; and (5) The age-friendly component of the projects. The final subsection deals with a short case study, which presents one the initiatives with an explicit age-friendly component (i.e., older people’s access health services), namely the 4D Cities project from Baia Sprie.

The findings suggest the existence of a process of initiating and implementing smart city initiatives in Romania, in which some factors play an important role. For example, already existing economic disparities between the seven Development Regions in Romania create the context of different opportunities to contribute as local municipalities with local funds in cofinancing such projects. In addition, disparities in the economic development of the different regions create a chain in the process of implementing smart cities projects that would work in favour of the more advanced and developed regions: the ability to attract major private companies to invest in public–private partnerships for such initiatives is not equally distributed, and the human resources as well are concentrated in the most developed regions.

**Figure 5:**
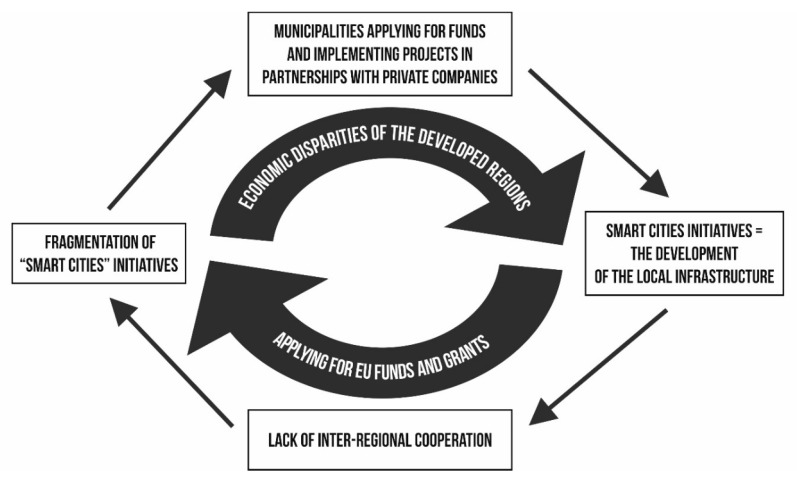
The vicious cycle of the regional disparities and transnational funding of the smart city projects.

Regional disparities and the differences in the economic development between Development Regions favour the development of smart city initiatives in the three most developed regions of the country: Ilfov Region, North-West Region and West Region, whereas the other regions are left behind. The process perpetuates the cycle of inequalities (Figure 5).

Other important players with a decisive role in the chain of the implementation of smart city projects in Romania are the transnational organisations, mainly European Union programmes and grants, which are almost an exclusive source of financing of such projects for local and regional authorities. Consequently, the local authorities (most of the time municipalities) submit projects in line with those grants and not necessarily in line with the local priorities. This leads to a second vicious cycle (Figure 5), namely a gap between the local needs in terms of enhancing some of the “smart city” ideas, for example, the increased access for vulnerable persons to services and city opportunities) and the transnational priorities reflected in the European Union grants. On the one hand, local authorities initiate projects that are granted by the transnational bodies (for example, the Economic Competitiveness Sectorial Operational Program) and this would give them expertise in applying and implementing such projects and moving the “smart city” agenda forward. On the other hand, other opportunities to launch such projects (for example, through regional cooperation) are not sufficiently explored.

The chain of disparities (Figure 5) shows a tendency towards top-down approaches in terms of funding, with the transnational organisations having a central role in the “smart city” priorities set by the local authorities. In addition, there is also a tendency towards fragmentation and a poor articulation between smart city initiatives, due to their local implementation.

### 4.1. The Regional Disparities and Lock on Inter-Regional Cooperation

Smart city solutions have been implemented in a few cities in Romania. The most important cities, in terms the number of citizens, the size of the industry and the economic power, are Bucharest (the capital city), Brașov, Sibiu, Timișoara, Craiova, and Cluj-Napoca. Most of the initiatives in these cities were local or regional initiatives, and the few national initiatives concerned mainly programmatic documents, policies, training activities or strategic action plans. Some of the national initiatives aimed to promote the smart city idea and smart city projects (such as the Annual Smart City Urban Projects Fair, since 2017).

Although the concrete smart-city actions were focused on the local and the regional levels, they accentuate the regional disparities at the country level, namely by be concentrated in the three most developed regions in Romania: the Bucharest-Ilfov Region, the North-West Region (around the cities Cluj-Napoca, Baia Mare and Oradea), the West Region (around the city of Timișoara), as well as in the Centre Development Region (around the cities of Brașov and Sibiu). The other four Development Regions (North-East, South-East, South-Muntenia, and South-West Oltenia) were less represented in regional initiatives and were represented more at the local level, as one-time and short-term projects. Only two large cities from the other four Development Regions were involved in smart-city initiatives in the 2012–2020 timeframes, namely Iași (located in the North-East region) and Craiova (located in the South-West Oltenia region).

This study also found that the regional economic disparities correspond with gaps in smart-city initiatives: more developed regions as the Bucharest-Ilfov Region and North-West Region have had more projects over the past eight years and have witnessed more articulated initiatives, whereas the other regions had fewer initiatives, saw more contextual projects (particularly related to calls for projects at the European Union level) and saw an actual lack of a long-term vision to the implementation of the “smart city” idea. Nevertheless, the already existing economic disparities between the Development Regions are reflected in the way smart cities projects were initiated and implemented. The lack of a strategic policy plan at the national level may eventually result in an increased regional gap.

In fact, the only regional cooperation that was found in the 30 analysed initiatives was the Western Alliance made up of the cities of Cluj-Napoca, Timișoara, Oradea, and Arad (Appendix A), launched in 2018, as a regional cooperation (North-West Region and West Development Region) aiming to improve the infrastructure in this part of the country. The agreement of the municipalities from the main cities in the two regions is focused on private–public partnerships through infrastructure projects, namely the support for the Transylvania Motorway project and Timișoara—Belgrade Motorway project, metropolitan underground transport systems, and tram projects, which are all positioned under the smart city umbrella (i.e., the ecological mobility). 

Some of the medium-sized cities, such as Suceava and Vaslui (North-East Development Region), manage to have two large projects in the domain of smart cities: Suceava between 2012 and 2015, and Vaslui, between 2014 and 2020. In both cities, the idea of urban markets and sustainable food in urban communities (low carbon emissions) exploited the specificity of the region, such as the rural population, the presence of small farms, and an ageing/older population which is highly affected by emigration. 

### 4.2. The Governance

Smart city solutions were only implemented in a few cities in Romania. With the exception of The National Strategy for Smart City, a programmatic document, the rest of the initiatives are private projects which with a mix of public–private governance: private companies in partnership with public authorities, most of the times municipalities, city halls, local authorities, but also national authorities such as The Ministry of Regional Development and Public Administration, and The Ministry of Energy. In the two operational programmes, Regional Operational Programme (version 1 for 2007–2013 and version 2 for 2014–2013), research institutes and universities were also involved. The role of private operators and the role of the industry, in general, seem to be very important in all the initiatives. Additionally, the number of economic operators is relatively large. For instance, over 600 economic operators contributed to the Regional Operational Programme. The industry participates in the activities and implementation stage of various programmes, as well as in training activities, in elaborating policies and action plans and in promoting different programmes related to the smart city concept. For example, The Romanian Association for Smart City and Mobility (RSCMA), a private body, forms a working group and takes part in the Smart City National Strategy. In addition, the RSCMA runs the Smart City Academy, a national training platform. The mission of this platform is to train smart city experts working for the participating companies, as well as from the central and local administrations. Moreover, as many activities from the smart city programmes in Romania consist of implementing digital solutions, Internet-based applications, free Wi-Fi in public transportation, and Internet-based technical solutions, the large multinational enterprises already existing on the Romanian market played a key role in the smart city initiatives. For example, in the Smart City Alba Iulia 2016–2018 pilot project, two large telecommunications’ companies, which are key actors in the Internet and telephony industry on the Romanian market, played an important role. Additionally, some private companies offered developing grants, for example, in the case of Bucharest and the Smarter Cities Grants.

The role of the private enterprises in leading or implementing smart city projects in Romania comes along with accusations of corruption and potential distrust of the citizens in the partnership between the local administration (such as municipalities) and the industry. For example, Bucharest Smart City Development Strategy was based on a partnership between the Bucharest City Hall and an accountancy firm—in which the private enterprise provided consultancy in defining the strategy; this project was discussed in the local media because of alleged corruption [33]. Nevertheless, the partnership between private and public institutions is a relatively common aspect in all the smart city initiatives that were analysed for the time frame of 2012 to 2020.

### 4.3. Type of Projects and Main Objectives

Among the 30 analysed initiatives two were programmatic documents: Romanian Smart City Projects (a strategic plan started in 2016, aiming at the implementation of 320 Smart City projects in Romania, with a budget of €15 billion); and The National Strategy for Smart City (2018) developed under the supervision of The Ministry of Regional Development and Public Administration of Romania and experts from other ministries, including a working group from RSCMA. Both are strategic action plans, with a long-term approach. Three initiatives aimed to promote projects in the area of smart city concepts, and its activities were linked to the idea of promotion and awareness: Smart City Industry Awards (since 2016); Smart City Caravan (since 2017); Annual Smart City Urban Projects Fair (since 2017). Besides promotion, complementary projects have been developed, having publishing (Smart City Magazine, since 2016), training of experts (Smart City Academy, since 2017) and respectively lobbying (The Romanian Association for Smart City and Mobility working group, 2018) as objectives. Additionally, some of the initiatives remained in the stage of policies and strategic action plans, without any concrete actions or implementation (at least not in the mentioned initiative): Baia Mare USE ACT Urban Renewal (2015-policy plan); Bucharest IBM Smarter Cities Challenge grant (2010–2011, strategy and business analytics); Alba Iulia: City Logo (2010, a branding initiative focused on cultural tourism). The latter one was a contextual project aiming for a revival of the historical city of Alba Iulia, in anticipation of the 2018 centenary celebration of Romania becoming a unified state (The Great Union of 1918, at 1st December, Alba Iulia). Practically, one-third of the initiatives that were analysed were not followed by actual actions and implementation.

The rest of the initiatives required different actions and implementation of the various strategic plans. The main objectives of the actions (Appendix A) were coded, and it revealed that the smart city concept sphere is reduced to the following aspects: smart transport and ecological mobility (six initiatives); Free Wi-Fi and public safety using Internet solutions (six initiatives), recycling and garbage collection, and lower CO_2_ emissions (six initiatives); saving energy including the control of lighting and public illumination (five initiatives); e-government (four initiatives), regeneration of small and medium-sized towns and disadvantaged neighbourhoods (four initiatives); sustainable development and land use (four initiatives); intelligent buildings and renewable and sustainable energy (four initiatives); and smart tourism such as a city app (four initiatives). The main objectives of the action plans in the different cities and small localities were focused on improving the infrastructure by applying ecological aspects and Internet-integrated applications. There was no particular regard to improving the daily lives of different categories of the Romanian population (such as older people or even peasants) that might be at risk of exclusion and marginalisation in the new “smart” living environment.

### 4.4. The Role of Transnational Organisations

The strategic programmes on smart cities have been initiated in Romania in close connection with programmes run by the European Union (Appendix A). Dedicated transnational bodies (for instance, European Union programmes and grants) played a key role in what type of projects was run through the country and which time frame they had. Particularly, The Smart City Association and Economic Competitiveness Sectorial Operational Program had a role in sustaining, financing, and promoting four of the analysed initiatives, and also the Economic Competitiveness Sectorial Operational Program (2007–2013; 2014–2020) supported some of the initiatives as a part of their grants. The same is valid for the Regional Operational Programme 2014–2020, which was also initiated by the European Union, who had a key role in the projects developed by the Bucharest Municipality and Bucharest-Ilfov Development Region. The European Union programmes created the opportunity of applying for grants in the smart city area, but also guidelines and standards of quality. The programmes also indicated specific smart city objectives, leaving some freedom to the regions, while prioritising some of the domains (as, for example, infrastructure and Internet-based technologies). In addition, the European Union programmes have contributed to fragmentation, as many of the projects run exclusively within the specific time frame of a specific European Union programme.

One important organisation, which plays a role in many of the actions concerning smart city initiatives, is URBACT (www.urbact.eu), a European Union organisation that promotes and monitors urban projects in the European Union member states. URBACT has a specific mission to help cities (including by financing programmes) in order to create sustainable development, by integrating environmentally friendly solutions. Seven out of the 30 analysed initiatives were supported, financed, and/or promoted through URBACT and this explains the focus of the programmes on the main objectives listed above. In addition, some private organisations have financed smart city initiatives. Still, the role of such private organisations in financing important, long-term strategies in the area of smart cities remains rather peripheral. Such enterprises are mostly involved in private–public partnerships with local municipalities or city halls.

### 4.5. The Age-Friendly Component

The age-friendly component is mostly absent in the most prominent smart city programmes and strategies in Romania for the past eight years. Still some initiatives (six out of 30) have an implicit age-friendly approach, as some of the initiatives address older people as a potential target group. For example, the RE-Block Disadvantaged Neighbourhoods initiative (Iași, 2018) was coded as an initiative that implicitly addresses the issue of age-friendliness. The initiative addressed disadvantaged communities in Romania (in Iași county and beyond), which are highly depopulated, with large segments of the population (particularly older population from rural areas and small localities) living in poor conditions, and with a lower quality of life. However, when going through the objectives and the action plans, such issues were addressed implicitly; not in explicit measures directed to the vulnerable cohorts of the population. The six initiatives were coded as implicit age-friendly programmes because they were implemented in the less economically developed Development Regions, which are highly affected by emigration and which have an increasingly older population. Still, neither of these initiatives explicitly addressed the age-friendly component, nor included age-friendly objects in the action plans. By addressing the disadvantaged neighbourhoods, these initiatives had a more bottom-up approach and focused more on the disadvantaged local communities and their particular needs.

The lack of age-friendly components in the existing smart city initiatives in Romania is also linked with the type of programme objectives such initiatives had in recent years. As mentioned before, infrastructure, ecological aspects (such as recycling), smart transportation, and energy saving were among the most common priorities in the way “smart city” phenomena were operationalised during the past eight years. For instance, the project by the Western Alliance did not mention any age-friendly component as a goal of any trans-regional cooperation. The aforementioned projects in Suceava and Vaslui dealt with the idea of urban markets and sustainable food. These two cases are of interest because they had an implicit age-friendly component. In fact, only six out of 30 initiatives could be coded as having an explicit age-friendly component, all in relatively small-sized cities. Besides the two projects in Suceava and Vaslui, the project from Baia Sprie (4D Cities Baseline Study, Appendix A) is a project aiming to diversify the health services based on 4D tools, especially in emergency and safety situations. This project is used for the case study described below.

#### Case Study: Baia Sprie 4D Cities

The 4D Cities Baseline Study project (2012–2013; 2014–2020) was financed by URBACT, under the European Regional Development Fund of the European Union. The 4D Cities project aimed to promote innovative ways of delivering healthcare services and increase access to healthcare services of the vulnerable groups, including older people. Additionally, the projects aimed to stimulate social participation in providing innovative healthcare solutions (such as volunteering and crowdfunding), as well as for a public–private partnership involving the community in different action plans.

Baia Sprie is a small-sized town with 16,000 inhabitants, located 9 km from Baia Mare (a municipality and one of the largest cities of the North West Development Region of Romania). Traditionally, 50% of the local population worked in the mining industry and the percentage of people working has decreased over time, due to the closing of the local mines. The majority of the young population migrated to Baia Mare or moved abroad for work, whereas Baia Sprie became a typically small locality with an increasingly older population and poor living conditions. The high incidence of chronic diseases (as pulmonary diseases) is related to the miners’ working conditions, thus, the importance of the health services for the local population was underlined in the project. The closure of the Chronic Disease Hospital pressed the City Council of Baia Sprie to create a partnership with private healthcare providers. In this context, the 4D Cities Baia Sprie project aims to diversify the health services through community involvement (social innovation tools). The initial focus was on professional medical care for emergency and safety situations. Furthermore, the project included the restoration of a local health centre for post-traumatic rehabilitation and regular treatment. Older people were involved as beneficiaries of the project but not in the project design and implementation.

An URBACT local support group was created to work in partnership with the city hall and the health centre. Still, not much information is publicly available about the results of this project, the success indicators and its sustainability. The municipality gives the same information as the URBACT website, meaning that there is only access to the main objectives, the source of financing, and the strategic plan. Information regarding the implementation, the success indicators, the evaluation of the project, possibilities of continuation, difficulties, and risks and also about the community involvement is not publicly available.

## 5. Discussion

Consistent with Bătăgan [34] and Rotună et al. [35], who previously analysed the dynamics of various indicators of the smart city concepts in Romania (such as e-governance [34]; smart cities ranking indicators [35]), this study also found that the regional economic disparities correspond with gaps in smart-city initiatives: more developed regions as the Bucharest-Ilfov Region and North-West Region have had more projects over the past eight years and have witnessed more articulated initiatives, whereas the other regions had fewer initiatives, saw more contextual projects (particularly related to calls for projects at the European Union level) and saw an actual lack of a long-term vision to the implementation of the “smart city” idea. In short, they were developed mainly in a few large cities: Bucharest (the capital city), Brașov, Baia Mare, Cluj-Napoca, Iași, and Timișoara. Some of the small to medium-sized cities where such programmes have been deployed (such as Alba Iulia, Suceava and Vaslui) benefitted from contextual European Union programmes and these cities got involved in a one-time initiative aiming at the revival of potential historical or cultural heritage. Manika [36] also found that in the case of smart cities, the European Union public procurement legislative framework encourages the deployment of innovation and sets the scene for a more strategic procurement for smart cities. 

This study further found that smart city initiatives around the country accentuate the socioeconomic disparities between the eight Development Regions of Romania. For example, the South-Muntenia Development Region did not have a single smart city initiative within its borders. Four other Development Regions were hardly represented in the current sample of documents as well. Taking into account that The National Strategy for Smart City was launched in 2018, the regional disparities should have been considered when updating the strategy. Furthermore, only one initiative involving the cooperation between two Development Regions was identified. The transregional cooperation in such projects could be a goal to follow when planning the long-term strategy for smart cities in Romania.

Most of the smart city initiatives analysed had a mixed governance, consisting of public–private partnerships, usually between city halls or municipalities and different private enterprises. Costantino and Pellegrino [37] analysed how public–private partnerships have been adopted across the world and their characteristics, both in developed and developing countries, through a multiple case comparison methodology. The three most important aspects characterising a public–private partnership transaction are the risk transfer to the private partner, the use of private financing, and the use of private expertise and management skills. They found the same behaviour in their case studies in terms of risk transfer to the private party and use of private expertise and management skills in public–private partnership projects. Developed countries showed significantly greater use of private financing [37]. 

Although the key role of the industry in Romanian projects can be explained by taking into account the technical solutions required in the implementation of smart city projects, some of the initiatives, strategies, consultancy, policy documents, and training, could have benefitted more from the expertise of public research institutes. In only two out of 30 initiatives, research institutes and universities were involved. It was noted that some of the initiatives have created public debate concerning the potential risk of corruption and this might happen also due to the prominent role of various private operations in all the stages of smart city programmes, from the design stage all the way up to the stage of implementation. 

Schipper and Silvius [38] looked at the characteristics of smart sustainable city development and the implications for project management. They stated that cities may require a centralised and comprehensive approach to strike an appropriate balance between diverse service exploration in different domains and intensive service exploitation. Publicly driven partnerships may help to accelerate smart city adoption at an early stage [38]. In the case of Croatian smart cities, Milenković et al. [39] showed how the government’s role in public–private partnership projects is to evaluate and approve detailed execution plans of the concessionaire while the private partner’s role is to design, build, finance, and operate the facilities. 

The initiatives that were analysed for the Romanian context covered only some dimensions of the concept of a “smart city”, particularly smart transport and ecologically-friendly mobility, free Wi-Fi and public safety, recycling and garbage and lower CO_2_ emissions, energy savings, e-government, sustainable development and land use; regeneration of small and medium towns and disadvantaged neighbourhoods, intelligent buildings, renewable and sustainable energy, and smart tourism, which is in line with the societal aspects and key performance indicators of smart cities identified by other studies including Baltac [40] and Angelakoglou et al. [41]. Only one initiative was identified which addressed the access to services (health services) for vulnerable cohorts. Therefore, it is concluded that the “smart city” idea in Romania is strongly linked to the improvement of the country’s infrastructure, saving energy and reducing CO_2_ emissions, which is a relatively restrictive view of the concept of smart cities. The notion that some cohorts within the population, older people in particular, may benefit from the outcomes of some of the smart city programmes is not explicitly stated. The lack of age-friendly components in the Romanian smart city initiatives could be partially explained by the fact that local authorities have focused more on projects connected with the development of the infrastructure (for instance, infrastructure for recycling and saving energy), and to a lesser extent on the community needs. This is visible from the documents selected in the corpus and coded (Appendix A). The case of Romania could be typical for other middle-income countries in the European Union, as developing infrastructure, including Internet-based technologies, is seen as a sign of progress by the local authorities. Consequently, the smart city initiatives would not have a purpose in addressing people’s needs but in enhancing economic development and local infrastructure. Such aspects need to be further analysed in other Eastern European countries and investigate whether similar patterns are to be found. Any smart city approach in Romania focusing on Internet-based technologies as a way of increasing people’s quality of life may be a first step in connecting the agendas of the smart cities and the age-friendly cities movements. For instance, in the case of improving the energy efficiency of homes of older people, there could be an explicit connection between smart home technology to combat fuel and energy poverty among senior citizens [42]. In terms of environmental hazards such as floods or the protection of older people against climate extremes [43], both agendas may intersect. Here, the approach by Jiménez [44] could be of importance, who calls for new models of collaboration. Such models include collaboration with citizens, which leads the smart city communities beyond the approach of public–private partnerships. This should lead to a Quadruple Helix model defined by Public–Private–People Partnership or PPPP, in which citizens have a say about the design of solutions for their city. There are some positive exceptions, namely in projects conducted in small or medium-sized localities as Suceava, Vaslui, and Alba Iulia. In these cases, the initiatives are connected to the local potential (for example, the sustainable food and the renewal of the urban markets in Vaslui and Suceava). However, such initiatives are scarce and mostly contextual. The full potential of connecting the two agendas has not been achieved. The case of Romania could be typical for other middle-income countries in the European Union, as developing infrastructure, including Internet-based technologies, is seen as a sign of progress by the local authorities. Consequently, the smart city initiatives would not have a purpose in addressing people’s needs but in enhancing economic development and local infrastructure. Such aspects need to be further analysed in other Eastern European countries and investigate whether similar patterns are to be found.

The strategic programmes in the domain of smart cities have been initiated in Romania in close connection with programmes run by the European Union. One important European Union organisation that promoted many of the actions of the smart city initiatives is URBACT, but also the sectorial operational programmes at the European Union level played an important role in dictating what type of initiatives were deployed, and which activities and main objectives to follow. Consequently, this study acknowledges the fact that European Union programmes and bodies played a key role in smart city strategies and projects developed in Romania during the past eight years. It also acknowledges that municipalities adjust their plans to apply for projects, and set their goals to fit the main programmes launched by the transnational organisations, without setting their own priorities for local development. The European Union rhetoric is in favour of developing infrastructure, recycling projects, renewable energy and sustainable transportation for a new member state as Romania. Therefore, municipalities are trying to fit into such rhetoric. European Union programmes are often seen as “the only alternative” for developing long-term initiatives at the regional level [45]. The risk, in this case, is to neglect other opportunities for financing and the role of the local resources, and to priorities some domains that are not in line with the local priorities and to pay little attention to inter-regional cooperation—an aspect that was already discussed.

Finally, the age-friendly component is missing in the analysed initiatives. In only six out of 30 analysed documents, the age-friendly component is implicit, such as the revival of small to medium-sized localities and the notion of improvements to disadvantaged neighbourhoods. In only one initiative, there was an explicit reference to the older people’s access to the local health services and smart city solutions in order to increase access to, and the quality of, health services. In the future, the agendas for the further development of the age-friendly component in smart cities in Romania could start by connecting one or more of the eight domains of an age-friendly city, namely Social participation; Communication and information; Civic participation and employment; Housing; Transportation; Community support and health services; Outdoor spaces and buildings; Respect and social inclusion, to the strategic plans and programming of smart city initiatives. The new smart age-friendly ecosystem framework [9] could provide further guidance for a connection between both agendas. Given the nature of the smart city projects in Romania, a connection between the domains of Transportation and Housing could be a logical first step. For instance, the creation of healthcare smart homes [46,47], and the utilisation and deployment of ICT and networked technological solutions [11,12,13,48], with a specific focus on older people as a target group, would be concrete actions that the Romanians could engage in. As stated before, sufficient and adequate training for older users could be a key requisite. Co-creation, co-design and a structured approach to stakeholder involvement could be a stimulus for the active involvement of older people in the decision-making concerning age-friendly and smart cities [49,50]. Additional guidance could be found in the works by Peine and Neven [51,52], which could help overcome suboptimal investments by policy-makers and companies when failing to create scale and impact in the domain of older people and technologies. 

One important issue that needs further analysis is the fact that the initiatives that were analysed in the present study are not transparent in the type of information they share with the general public. Even though these initiatives are run by public bodies (municipalities or the local authorities), they should have been more transparent in terms of the output achieved, as they are publicly accountable. Data regarding the success indicators of the action plans, risk evaluations, possibilities of continuation and the sustainability of those projects could not be found. Therefore, it is not possible to say how successful these initiatives were in achieving the initial goals and whether these initiatives have created a significant improvement in the lives of the communities and particularly in the lives of older people.

## 6. Conclusions

This study used the methodology of document analysis in order to investigate the characteristics of smart city initiatives developed in Romania over the past eight years (2012–2020). Such projects were often located in the Bucharest-Ilfov Region and the North-West Region. Smart city initiatives around the country accentuate the socioeconomic disparities between the eight Development Regions of Romania. Most of the smart city initiatives consisted of public–private partnerships. The initiatives covered only some dimensions of the concept of a “smart city”. Only one initiative was identified which addressed the access to services (health services) for vulnerable cohorts. The “smart city” idea in Romania is strongly linked to the improvement of the country’s infrastructure and sustainability, which is a rather restrictive view of the concept of smart cities. The notion that older people may benefit from the outcomes of some of the smart city programmes is not explicitly stated. In contextual projects conducted in small or medium-sized localities, there are age-friendly components to be identified. The full potential of connecting the agendas of smart cities and age-friendly cities, however, has not been achieved in Romania to date.

The case of the smart city initiatives presented here could be described as a typical case for the developing countries in the European Union. Though we can extend the conclusions of our research to other Eastern European countries, the mechanisms described in the findings have the potential for generalisation. Limitations of the current research lie in the absence of a comparative dimension, for example, in exploring what type of small cities projects have been developed in other European Union member states, in the new member states as compared to the old members states and what factors are regulating the chains of implementation of such projects in countries other than Romania. Still, Romania can be considered as a typical case, at least for the new European Union member states, as the mechanisms suggested here are largely influenced by the opportunities to get funding through European Union programmes.

Additionally, the relative peripheral role of the age-friendly component in the smart city initiatives identified in the analysis might not be a feature typical of the Romanian context. In the absence of similar analyses on other Eastern European countries, the factors that lead to neglecting of the age-friendly component can only be speculated about. The condition that Eastern European countries have experienced a serious increase in the older population only during the last 20 years, the neglect of age-friendly cities by the national governments in long-term strategies and policies, and also the fact that those countries have been prioritising the development of infrastructure as an indicator of success for the local governance, may be part of the explanation.

## Figures and Tables

**Table 1:** Basic statistics of Romania [25,26].

Total Population (2017)	19,778,000
Gross national income per capita (EUR PPP international $, 2017)	18,800
Life expectancy at birth m/f (years, 2017)	72/79
Probability of dying under the age of five (per 1000 live births, 2018)	7
Probability of dying between the ages of 15 and 60 years m/f (per 1000 population, 2016)	191/77
Total expenditure on health per capita (Intl $, 2017)	1079
Total expenditure on health as % of GDP (2017)	5.0

**Table 2:** The population of Romania and the main Development Regions [26].

	Total Population 1992	Total Population 2019
Romania	23,143,860	22,204,507
Urban Romania	12,478,618	12,520,160
Rural Romania	10,665,242	9,684,347
North-West Region	2,964,507	2,833,789
West Region	2,118,807	2,003,368
Centre Region	2,709,383	2,631,033
North-East Region	3,861,059	3,979,271
South-East Region	3,000,498	2,828,048
South-West Region	2,448,573	2,163,319
South Region	3,574,993	3,194,237
Bucharest and Ilfov Region	2,466,040	2,571,442

**Table 3:** The older population of Romania per age cohort [26].

Age Range	Romania		Urban Romania		Rural Romania	
	1992	2019	1992	2019	1992	2019
65–69	1,033,959	1,251,318	403,076	755,541	630,883	495,777
70–74	560,805	840,746	215,436	454,052	345,369	386,694
75–79	481,408	664,299	178,586	330,687	302,822	333,612
80–84	306,882	526,865	108,831	249,097	198,051	277,768
85+	141,672	389,476	49,506	185,201	92,166	204,275
Total	2,524,726	3,672,704	955,435	1,974,578	1,569,291	1,698,126

**Table 4:** The older population of main Romanian cities per age cohort [26].

Age Range	Cluj-Napoca		Constanţa		Brașov		Iaşi		Bucharest	
	1992	2019	1992	2019	1992	2019	1992	2019	1992	2019
65–69	11,033	19,728	10,938	22,422	10,400	20,494	8825	21,898	97,569	134,723
70–74	6902	13,349	5138	13,345	5739	11,781	4811	12,516	56,886	84,278
75–79	4409	10,110	4239	10,432	3911	8822	3711	8199	46,845	58,339
80–84	3295	7223	2593	7886	2524	7174	2450	6338	28,705	49,430
85+	1493	5504	1260	5591	1068	5240	1261	4558	12,891	49,211
Total	27,132	55,914	24,168	59,676	23,462	53,511	21,058	53,509	242,896	375,981

## Appendix A

**Table A1:** The corpus of analysis and the results of the coding process.

No Crt.	Name of the Initiative	Year	Initiated by	Type of Action	Governance	Documents/Activities/ Training/Promotion	Main Objectives	Age-Friendly Component?	Role of the Industry	Role of a Transnational Body/Organisation
1	Smart City Alba Iulia 2016–2018 pilot project	Since 2018	Regional Operational Program and local administration	Local	Private	Activities as part of a programmatic document	Energy consumption has been streamlined; local government controls the intensity of light; Buses with Wi-Fi, monitor air, projects to digitise education, and interact with public institutions	No	Telecom and Internet operator	Smart City Association and Economic Competitiveness Sectorial Operational Program, EU
2	Annual Smart City Industry Awards	Since 2016	RSCMA, High Patronage of the Ministry of Energy and local decision-makers; Ministry of Energy, Ministry of Waters and Forests, Chamber of Commerce and Industry of Romania	National	Private in partnership with public	Promotion	Awards and promotion	No	Key role	Idem above
3	Smart City Caravan	2017	RSCMA	National	Private in partnership with public	Promotion	Promotion of smart city solutions and education of decision-makers on new technologies, run in 12 regions of Romania.	No	Private association organising smart city courses	Idem above
4	Annual Smart City Urban Projects fair	Since 2017	RSCMA	National	Private in partnership with public	Promotion	Projects/fair-promotion	No	Key role	Idem above
5	Work group for Smart City National Strategy 2018	2018	RSCMA	National	Private in partnership with public	Documents	Lobby	National Strategy for Smart City does not have an explicit component	Key role	Not specified
6	Smart City Magazine	Since 2016	RSCMA	National	Private	Documents	Publication	No	Key role	Idem above
7	Smart City Academy, National training platform	Since 2017	RSCMA	National	Private in partnership with public	Training	Training; The mission of this platform is to train Smart City experts for both central and local institutions as well as companies that are active in the development of cities and communities in Romania.	No	Key role	Idem above
8	Romanian Smart City Projects	Since 2016	RSCMA, High Patronage of the Ministry of Energy and local decision-makers; Ministry of Energy, Ministry of Waters and Forests, Chamber of Commerce and Industry of Romania	National	Private in partnership with public	Programmatic documents	320 Smart City projects in Romania (€15 billion available: 8 billion from national funds, 7 billion from EU funds)	No	Key role	Not specified
9	IASI Smart City	2016–2019	Municipality	Local	Private in partnership with public	Activities as part of a programmatic document	Smart transport and ecological mobility	Not specified	Key role	EU program
10	Cluj-Napoca Smart City	2013–2019	Municipality	Local	Private in partnership with public	Activities as part of a programmatic document	Smart transport and ecological mobility	Not specified	Key role	EU program
11	Cluj-Napoca – Timișoara – Oradea – Arad- the Western Alliance	Since 2018	Municipalities in the mentioned cities	Regional Region West and North-West	Public	Activities as part of a programmatic document	Development of motorway/road, trains, and air traffic infrastructures. The increase in ecological mobility through public transportation. Support of the public–private partnership through infrastructure projects	No	300 industry operators	No
12	www.e-guvernare.ro, Bucharest	2013	Bucharest City Hall	Regional-Region Bucharest-Ilfov	Private in partnership with public	Activities as part of a programmatic document	e-governance	No	Key role	No
13	Regional Operational Program EU	2007–2013	Region Bucharest-Ilfov and (Bucharest, Brasov, Sibiu, Timișoara, Craiova and Cluj-Napoca)	Regional, and large cities	Private in partnership with public	Activities	To create video T systems for areas with high criminality and to rehabilitate the public lighting system	Implicit component	Key role, 600 economic operators and research institutes and universities	Economic Competitiveness Sectorial Operational Program, EU
14	Regional Operational Programme, EU	2014–2020	Region Bucharest-Ilfov and Bucharest, Brasov, Sibiu, Timișoara, Craiova and Cluj-Napoca	Regional, and large cities	Private in partnership with public	Activities	Territorial cohesion in the urban regeneration of small and medium towns, especially those mono-industrial.	Implicit component	Key role, 600 economic operators and research institutes and universities	Idem above
15	Timișoara Smart City	2007–2013	Local municipality, Vest Region, Polytechnic University Timișoara	Local	Private in partnership with public	Activities	Smart transport, adaptive control system for traffic management, video surveillance	Implicit component	Key role	Idem above
16	Sibiu. ECRO, The Smart City Sibiu Project	2012–2015	Local municipality	Local	Private in partnership with public	Activities	Smart grid concept (energy efficiency and the reduction of CO_2_ emissions)	No	Key role	Idem above
17	Craiova. Craiova Smart City. ARCA	2018–2021	Local municipality. Ministry of Energy	Local	Private in partnership with public	Activities	e-governance, digital public services, digital services (City app-for tourists)	No	Key role	EU POCA program
18	Brașov, Smart City	Since 2008	Local municipality. Ministry of Energy	Local	Private in partnership with public	Activities	Telemetry system for public lighting (saving up to 35% during nighttime); intelligent containers for garbage and recycling	No	Key role	URBACT, EU. Other EU program
19	Alba Iulia: City Logo, Metropolitan Governance	2010	Alba Iulia municipality and URBACT, RO. The Ministry of Regional Development and Public Administration of Romania	Local	Private in partnership with public	Activities promotion, branding, action plan, local governance	A branding initiative focused on cultural tourism	No	Key role	URBACT, EU
20	Iași. RE-Block Disadvantaged Neighbourhoods	2018	Iași municipality and URBACT, RO. Ministry of Regional Development and Public Administration of Romania	Local	Private in partnership with public	Activities	Foster efficient regeneration of disadvantaged neighbourhoods’ environmental quality, green neighbourhoods	Implicit component	Key role	URBACT, EU
21	Baia Mare USE ACT	2015	Baia Mare municipality and URBACT, RO. The Ministry of Regional Development and Public Administration of Romania	Local	Private in partnership with public	Documents and policies	Sustainable development and land use policy	No	Not specified	URBACT, EU
22	Baia Sprie. 4D Cities	2012–2013; 2014–2020	Baia Sprie local administration and URBACT, RO. The Ministry of Regional Development and Public Administration of Romania	Local	Private in partnership with public	Strategy, action plan	Diversifying the health services: professional medical act in emergency and safety situations, based on innovative tools	Explicit component	Not specified	URBACT, EU
23	Suceava. Urban Markets Renewal	2012–2015	Suceava municipality and URBACT, RO. The Ministry of Regional Development and Public Administration of Romania	Local	Private in partnership with public	Strategy, actions	Sustainable urban markets	Implicit component	Key role	URBACT, EU
24	Vaslui, Urban Markets. Sustainable Food in Urban Communities, Low Carbon Emissions	2014–2020	Vaslui municipality and URBACT, RO. The Ministry of Regional Development and Public Administration of Romania	Local	Private in partnership with public	Strategy, actions	Development of a competitive, safe, and healthy local food system, with low CO_2_ emissions	Implicit component	Key role	URBACT, EU
25	Bucharest: Smarter Cities Challenge grants	2010–2011	Bucharest Municipality and IT international private Company	Local	Private in partnership with public	Strategy, analysis	Integrated Operations Centre and business analytics	No	The only role, private initiative	No
26	Urban regeneration and Regional Operational Programme in Bucharest-Ilfov Region	2014–2020	Bucharest Municipality and Bucharest-Ilfov Region	Regional	Private in partnership with public	Actions	Measures dedicated energy-efficiency for buildings and urban mobility; Increase Energy Efficiency;	No	Key role	Regional Operational Program 2014–2020, EU
27	Bucharest Smart City Development Strategy	2018	City Hall Bucharest and accountancy private company	Local	Private in partnership with public	Document, strategy, consultation	Traffic management, lighting, infrastructure, e-government, public safety, telecommunications, environment – intelligent buildings, green energy, smart tourism	No	Key role	No
28	Bucharest Smart City projects	2017–2019	City Hall Bucharest and private sector (Telekom Romania and Cisco)	Local	Private	Actions	Smart city space in the Youth Park in Bucharest	No	Key role	No
29	Bucharest Smart City Map	2017	City Hall Bucharest and ANAGRAMA, software company	Local	Private in partnership with public	Actions	Intelligent City Map Application and City Drop. Software solution Incident Report; Free Wi-Fi; Accessibility; Local Recycle – Recycle locally; City Tourism – Tourism in town	No	Key role	No
30	National Strategy for Smart City	2018	The Ministry of Regional Development and Public Administration of Romania, other Ministries	National	Public	Programmatic document	Long-term strategy	No	Consultancy	No specify
Links: Smart City Alba Iulia 2016–2018 pilot project: https://hub.beesmart.city/city-portraits/alba-iulia-how-central-romania-quietly-created-a-smart-city-champion-in-europe. Smart City Industry Awards Annual Awards: https://scia2018.romaniansmartcity.ro/ Smart City Caravan: https://caravana.romaniansmartcity.ro/ Annual Smart City Urban Projects fair: https://scup2018.romaniansmartcity.ro/ Work group for Smart City National Strategy 2018: https://businessforsmartcities.com/load/118/presentation/2_alexa_dimitrscu_1_28a81.pdf Smart City Magazine: https://smartcitymagazine.ro/ SMART CITY ACADEMY, national training platform: https://academiasmartcity.ro/ ROMANIAN SMART CITY PROJECTS: https://businessforsmartcities.com/load/118/presentation/2_alexa_dimitrscu_1_28a81.pdf IASI Smart City: https://iasismartcity.ro/ Cluj Napoca Smart City: https://romaniansmartcity.ro/cluj-napoca-locul-3-smart-city/ Cluj Napoca – Timișoara – Oradea – Arad- the WESTERN ALLIANCE: https://transylvanianow.com/four-romanian-cities-form-western-alliance/ www.e-guvernare.ro, Bucharest Regional Operational Program, EU Timișoara Smart City: https://mysmartcity.ro/ Timișoara Smart City: http://www.primariatm.ro/epress.php?epress_id = 8288 Sibiu ECRO, The Smart City Sibiu Project Craiova. Craiova Smart City. ARCA: https://caravana.romaniansmartcity.ro/craiova-implementeaza-proiecte-smart-city-caravana-smart-city Brașov, Smart City, https://romaniansmartcity.ro/brasov-smart-city-containere-inteligente/ Alba Iulia: City Logo, Metropolitan Governance https://urbact.eu/sites/default/files/alba_iulia_an_ancient_capital_.-local_action_plan.pdf Iași. RE-Block Disadvantaged Neighborhoods: https://urbact.eu/re-block-complete-overview Baia Mare. USE ACT. Urban Renewal: ttps://urbact.eu/sites/default/files/media/useact_lap_baia_mare_metropolitan_area.pdf Baia Sprie. 4D Cities: https://urbact.eu/sites/default/files/import/Projects/4D_CITIES/outputs_media/4D_Cities_Baseline_Study.pdf Suceava. Urban Markets Renewal: https://urbact.eu/sites/default/files/suceava_lap_long_version.pdf Vaslui, Urban Markets. Sustainable Food in Urban Communities, Low Carbon Emissions: https://urbact.eu/sites/default/files/sustainable_food_lap_summaries.pdf Bucharest. IBM Smarter Cities Challenge grants: http://www.smartercitieschallenge.org/city_bucharest_romania.html Urban regeneration and Regional Operational Program in Bucharest Ilfov Region. Regional Operational Program 2014-2020, EU Bucharest Smart City Development Strategy: http://2014-2020.adrbi.ro/media/3597/prezentare-smart-city_31-mai-2018.pdf Bucharest smart city projects: https://bucharestsmartcity.ro/ Bucharest Smart City Map – Intelligent City Map Application and City Drop: http://2014-2020.adrbi.ro/media/3082/bucharest-steps-to-a-european-smart-city.pdf National Strategy for Smart City: https://magnanews.ro/2018/07/strategia-nationala-smart-city/ URBACT, RO: https://urbact.eu/urbact-in-romania.										
